# Association between Chloroplast and Mitochondrial DNA sequences in Chinese *Prunus* genotypes (*Prunus persica, Prunus domestica, and Prunus avium*)

**DOI:** 10.1186/s12870-014-0402-4

**Published:** 2015-01-16

**Authors:** Tariq Pervaiz, Xin Sun, Yanyi Zhang, Ran Tao, Junhuan Zhang, Jinggui Fang

**Affiliations:** College of Horticulture, Nanjing Agricultural University, Nanjing, 210095 P R China; Institute of Forestry and Pomology, Beijing Academy of Agriculture and Forestry Science, Beijing, 100093 P R China

**Keywords:** Organelle DNA sequences, *Prunus*, SSR markers, Genetic diversity, *Prunus persica*, *Prunus domestica*, *Prunus avium*

## Abstract

**Background:**

The nuclear DNA is conventionally used to assess the diversity and relatedness among different species, but variations at the DNA genome level has also been used to study the relationship among different organisms. In most species, mitochondrial and chloroplast genomes are inherited maternally; therefore it is anticipated that organelle DNA remains completely associated. Many research studies were conducted simultaneously on organelle genome. The objectives of this study was to analyze the genetic relationship between chloroplast and mitochondrial DNA in three Chinese *Prunus* genotypes viz., *Prunus persica, Prunus domestica,* and *Prunus avium*.

**Results:**

We investigated the genetic diversity of *Prunus* genotypes using simple sequence repeat (SSR) markers relevant to the chloroplast and mitochondria. Most of the genotypes were genetically similar as revealed by phylogenetic analysis. The Y2 Wu Xing (Cherry) and L2 Hong Xin Li (Plum) genotypes have a high similarity index (0.89), followed by Zi Ye Li (0.85), whereas; L1 Tai Yang Li (plum) has the lowest genetic similarity (0.35). In case of cpSSR, Hong Tao (Peach) and L1 Tai Yang Li (Plum) genotypes demonstrated similarity index of 0.85 and Huang Tao has the lowest similarity index of 0.50. The mtSSR nucleotide sequence analysis revealed that each genotype has similar amplicon length (509 bp) except M5Y1 i.e., 505 bp with CCB256 primer; while in case of NAD6 primer, all genotypes showed different sizes. The MEHO (Peach), MEY1 (Cherry), MEL2 (Plum) and MEL1 (Plum) have 586 bps; while MEY2 (Cherry), MEZI (Plum) and MEHU (Peach) have 585, 584 and 566 bp, respectively. The CCB256 primer showed highly conserved sequences and minute single polymorphic nucleotides with no deletion or mutation. The cpSSR (ARCP511) microsatellites showed the harmonious amplicon length. The CZI (Plum), CHO (Peach) and CL1 (Plum) showed 182 bp; whileCHU (Peach), CY2 (Cherry), CL2 (Plum) and CY1 (Cherry) showed 181 bp amplicon lengths.

**Conclusions:**

These results demonstrated high conservation in chloroplast and mitochondrial genome among *Prunus* species during the evolutionary process. These findings are valuable to study the organelle DNA diversity in different species and genotypes of *Prunus* to provide in depth insight in to the mitochondrial and chloroplast genomes.

## Background

The *Prunus* (Rosacea, Subfamily Prunoideae) is a genus of small shrubs and trees, composed of five subgenera; including Prunopkora, Amygdalus, Cerasus, Padus and Laurocerasus that contain about 200 species [1,2]. Many species are economically valuable; especially species such as apricots, cherries, plums, peaches and almonds which are used as food and have ornamentals values [3]. Among these subgenera, Cerasus (Cherries) is considered to be the most diverse group. Although the members of subgenus Amgdalus like almonds and peaches are apparently related as they were hybridize early, but relatively distant from the member of subgenera prunopholal (plum and apricot) [4]. Wallien [5] assumed that *Prunus* is originated from central Asia. He also reported that plum species of subgenera prunophora are the central species for the evolution of the genus *Prunus*. Traditionally, nuclear DNA is used to assess the diversity and relatedness among different species, but since early 1980, variations at the DNA level of organelle genome has also been used to study the relationship among different species [6].

In most species of angiosperms, mitochondrial and chloroplast genomes are maternally inherited [7], therefore they are expected to be remained completely associated [8]. Chloroplasts (plastids) are plant organelles that contain small, self-replicating circular DNA, with highly conserved 130 genes with the size ranging from 72 to 220 kb [9,10]. The plant mitochondrial genome content is highly dynamic in its nature and is reported as the largest and the least gene-dense among eukaryotes [11]. Mitochondrial genomes of spermatophytes are the largest among all organelle genomes. Their large size has been attributed to various factors; though, the relative contribution of these factors to the expansion of mitochondrial DNA (mtDNA) remains undiscovered [12,13]. The mitochondrial genomes of seed plants are exceptionally variable in size, structure, and sequence content, with the accumulation and activity of repetitive sequences underlying such variation [14]. The plant mitochondrial genome content is highly dynamic: plant mitochondrial DNA (mtDNA) is the largest and least gene-dense among the eukaryotes and variable in size (200 to 2,500 kb), and contains many introns and repeated elements (typically 90% of the total sequence), [15,16]. Chloroplasts DNA (cpDNA) of green plants are exceptionally conserved in their gene content and organization that provides sufficient information for genome-wide evolutionary studies. The cpDNAs have been set as targets among the very early genome sequencing projects because of their small sizes [17,18]. Chloroplast DNA sequences are of great interest for population genetics and genetic diversity studies [19]. The genomic DNA sequences are valuable for resolving the plant phylogeny at deep levels of evolution because of their lower rates of silent nucleotide substitution [20].

The genomic studies concerning fruit species have been tremendously increased to characterize and analyze genetic diversity and conservation of fruit species germplasm resources; based on morphological characteristics and molecular markers [21-23]. Previously, SSR markers have been extensively used for molecular characterizations and detection of similarity relationships among *Prunus* genotypes. The results have revealed high polymorphism levels that discriminate the accessions [24,25]. Furthermore, structural characters in cpDNAs, such as gene order/segment inversions, expansion/contraction of genes, and expansion/contraction of the inverted repeat (IR) regions can serve as powerful markers for phylogenetic inference [20]. For the crop improvement, researchers usually studied genetic diversity among materials [23]. Plant cpDNAs have been set as targets among the very early genome sequencing projects owing to their small sizes [26]. Recently, the cpDNAs sequenced at least 200 plants have been completed (http://www.ncbi.nlm.nih.gov/genomes/GenomesGroup.cgi?taxid=2759&opt=plastid), and the numbers are rapidly increasing due to an extensive application of the second-generation sequencing technologies to the whole chloroplast genome sequencing [27]. Non-coding regions of cpDNA have been explored under this assumption that these regions should be under less functional constraint than coding regions and should provide greater levels of variation for phylogenetic analyses [28]. Many reports have proven their potentials in resolving phylogenetic relationships at different taxonomic levels and understanding structural and functional evolution by using the whole chloroplast genome sequences [17,26,29]. Additionally, concatenating sequences from many genes may overcome the problem of multiple substitutions that cause the loss of phylogenetic information between cp lineages [30]. However, there are few studies describing the association between the two organelle genomes in angiosperms [31-33]. In this study, three *Prunus* species peach, plum and pear were analyzed. The main objectives were to study the extent of organelle DNA sequence conversing levels, genetic diversity, phylogeny and genetic similarity and to investigate genetic relationships between cpDNA and mtDNA within and among *Prunus* species. Therefore, the present study will provide a proximal knowledge and justification for the low substitution rate of plant cpDNA and mtDNA, namely the existence of efficient recombination-associated and DNA repair activities.

## Results

### Phylogenetic analysis

**Figure 1:**
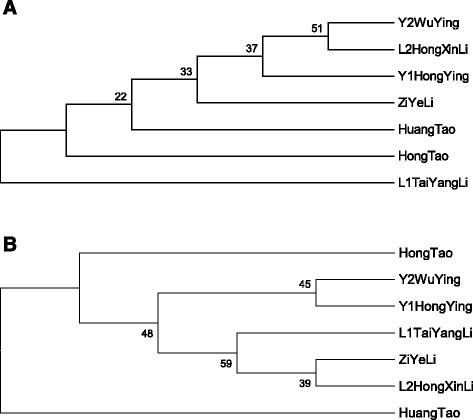
**Dendrogram of 7** ***Prunus*** **genotypes based on mtSSR markers. (A)** CCB256 **(B)** NAD6.

A phylogenic tree was constructed according to mitochondrial SSR data of 7 genotypes (Figure 1A and B). A close genetic similarity was detected among all genotypes ranging from 0.35 to 0.95. The prime CCB256 depicted that cherry (Y2 Wu Ying) and plum (L2 Hong Xn Li) have a high similarity index (0.89), whereas plum (Zi Ye Li) showed relatively closer similarity value of 0.85. In addition plum (L1 Tai Yang Li) has the lowest genetic similarity (0.35) as compared with the rest of the genotypes. These results suggested that cherry (Y1 Hong Ying) have a close relationship to Huang Tao than to Hong Tao (Peach genotypes) (Figure 1A). In case of second primer NAD6, Cherry genotypes (Y1 Hong Ying and Y2 Wu Ying) and plum genotypes (L2 Hong Xn Li and Zi Ye Li) showed close similarity with 0.89 similarity index (Figure 1B), while Huang Tao (peach) showed the lowest similarity index i.e., 0.52.

**Figure 2:**
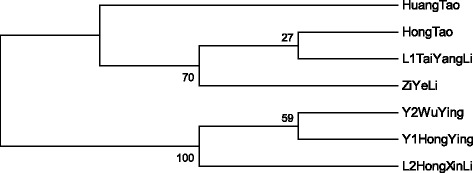
**Dendrogram of 7** ***Prunus*** **genotypes based on cpSSR marker.**

The cpSSR dendrogram was constructed (Figure 2) according to sequence data of single primer ARCP511. The results revealed from the phylogeny that peach (Hong Tao), plum (L1 Tai Yang Li) and cherry genotypes (Y2 Wu Ying and Y1 Hong Ying) have the similarity index of 0.85. While Huang Tao (peach) was less similar (0.50) and Zi Yeli (plum) was comparatively closer (0.68) to Hong Tao (peach) and L1 Tai Yang Li (plum) genotypes. Whereas L2 Hong Xn Li has the lowest similarity index in this cluster with a value of 0.68. From the phylogeny tree, it can be predicted that Hong Tao and L1 Tai Yang Li and Y2 Wu Ying and Y1 Hong Ying are closely related genotypes while Huang Tao and L2 Hong Xn Li showed the lowest similarity of 0.50 and 0.68, respectively.

The majority of genotypes placed in groups were found to have a high similarity index. The dendrogram showed that some genotypes in mtSSR and cpSSR have different genetic characteristics. For example, for the mtSSR sequences, with a similarity index of 0.85, Y2 Wu Ying, L2 Hong Xn Li, Y1 Hon Ying and Li Zi Yeli were the closest genotypes. However, L1 Tai Yang Li and Huang Tao have the lowest similarity with both primes of mtSSR. Although, in case of cpSSR sequences the closest genotypes are Hong Tao, L1 Tai Yang Li, Y2 Wu Ying and Y1 Hon Ying with a similarity index of 0.85, while Huang Tao and L2 Hong Xn Li were divergent genotypes.

### Nature of the polymorphism based on sequencing alignment

**Figure 3:**
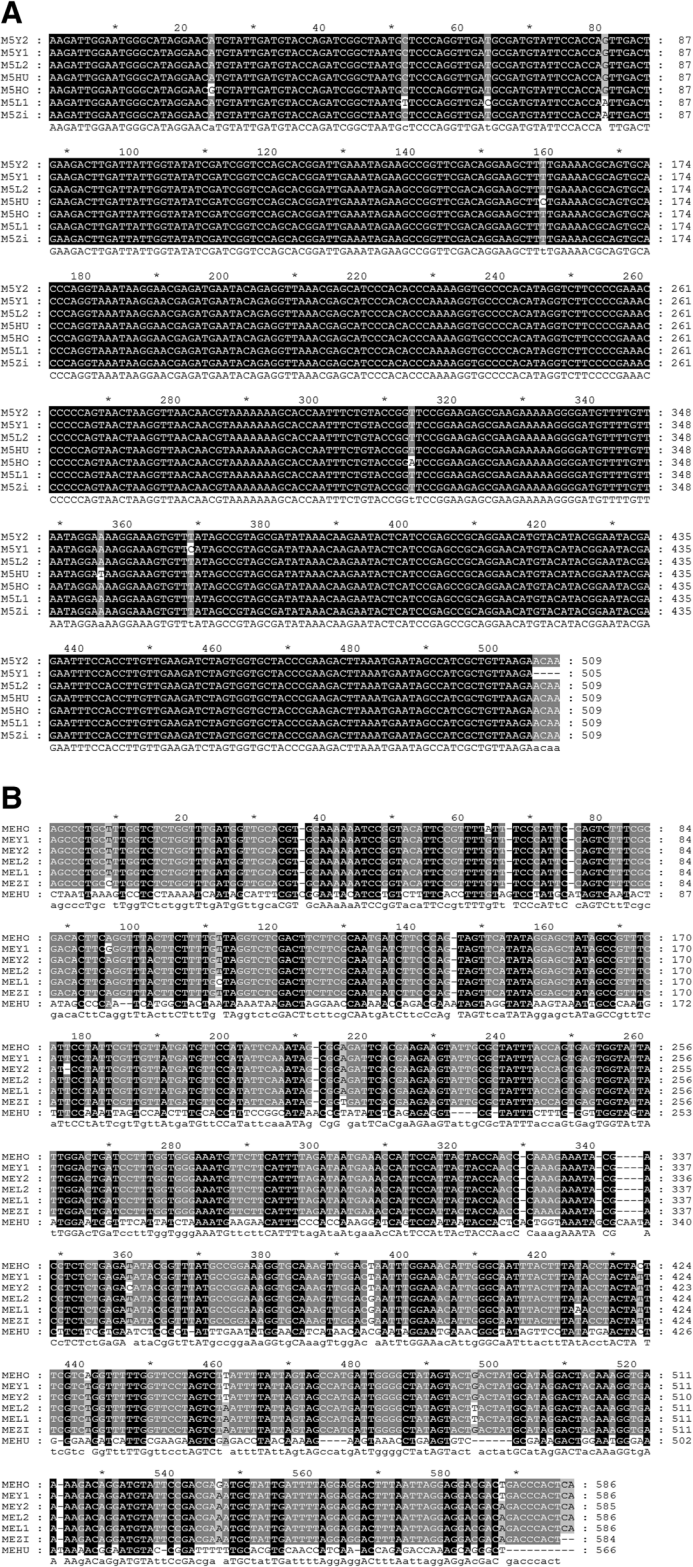
**DNA sequence alignment of allelic variants of mtSSR in** ***Prunus*** **genotypes.** Alignment of CCB256 **(A)** and NAD6 **(B)**. *M5 (primer CCB256), M5Y2 (Y2 Wu Ying), M5Y1 (Y1 Hong Ying), M5L2 (L2 Hong Xin Li), M5HU (Huang Tao), M5HO (Hong Tao), M5L1 (L1 Tai Yang Li), M5Zi (Zi ye Li).

The mtSSR nucleotide sequence analysis revealed that each genotype has similar (Figure 3A and B) amplicon length (509 bp) except M5Y1 having 505 bp with CCB256 primer; In case of NAD6 primer (Figure 3A), all genotypes showed different amplicon sizes, i.e., MEHO, MEY1, MEL2 and MEL1 has 586 bps; meanwhile MEY2, MEZI and MEHU have 585, 584 and 566 bps, respectively. The CCB256 primer showed highly conserved sequences and very few single polymorphic nucleotides were observed. The M5Y1 depicted SNPs at 369 and 356, M5HU at 160, M5HO at 25 and 314, M5L1 at 52 and 64 positions. There was no deletion, while many of them showed single important conserved sequence of 81 bp. All genotypes have G nucleotide except M5L1 and M5ZI (A). The MEHU was highly diverse compared to the rest of genotypes which showed the distinct relation between studied *Prunus* samples.

It was revealed from the analysis of NAD6 (mtSSR) (Figure 3B) that MEHU is highly polymorphic and having very less conserved sequences as compared to the rest of genotypes. The MEY1 showed SNPs at 96 and 588, MEY2 at 360, MEHO at 434, 441, 547 and 588, MEL1 at 112 and 425, MEZI at 9 and 217 bps.

**Figure 4:**
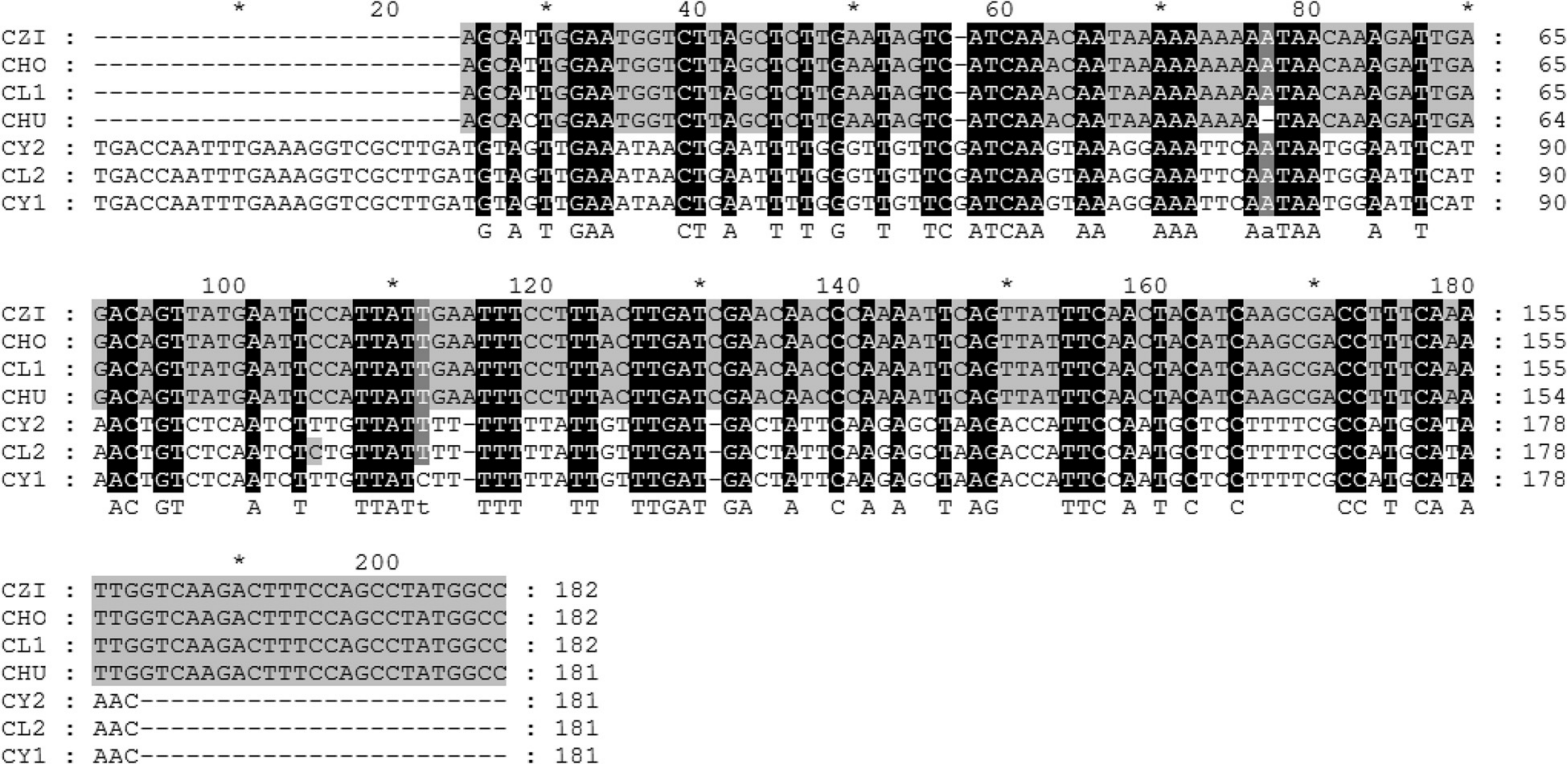
**Product sequences alignment of cpSSR marker in** ***Prunus*** **genotypes.** *C (primer ARCP511), CY2 (Y2 Wu Ying), CY1 (Y1 Hong Ying), CL2 (L2 Hong Xin Li), CHU (Huang Tao), CHO (Hong Tao), CL1 (L1 Tai Yang Li), CZi (Zi ye Li).

The cpSSR (ARCP511) microsatellites showed harmonious amplicon lengths. The CZI, CHO and CL1 had 182 bps and CHU, CY2, CL2 and CY1 showed 181 bp amplicon lengths (Figure 4). The deletions were observed at different positions e.g., for CZI, CHO, CL1 and CHU at position 57, for CHU at position 77 and for CY2, CL2 and Cy1 at position 131 (Figure 2). A high level of conservation was found among the studied genotypes that did not show species specific alleles.

In both SSR primers, sequence alignments of the cloned genotypes products suggested that microsatellite derived polymorphisms existed in both mitochondrial and chloroplast SSR allelic loci. The cpDNA was highly conserved and consequently polymorphism in most of mtSSR and cpSSRs was detected and a typical continuous pattern of variations in lengths that was most probably caused by the presence of variation in mononucleotide repeats. The cloned sequence alignments of the amplified products revealed that a variable poly (T) has a direct link to the polymorphism. The mtDNA polymorphisms observed in all genotypes studied with both pairs of primers showed the phylogenetic association among the genotype that might be useful for the preservation of *Prunus species*. The results of genotypes exhibits discontinuous array of allele sizes and repeated alignment of the duplicate sequences of its amplified products proved that both mitochondrial and chloroplast segments of DNA deletion or insertion were the major source of polymorphism.

### Sequence comparison between and among mtDNA and cpDNA

The mtDNA sequences were searched in the NCBI database using BLASTx tool. The results showed minimal overlaps with either mitochondrial genes or with mitochondrial exons of mitochondrial origin, including introns from the calculation of the mtDNA and cpDNA fractions assumed to represent nuclear imports. The BLAST search showed three different proteins including Cytochrome C assembly protein, PRKO 6433 super-family and ndhk in mitochondria and chloroplast, respectively. These proteins have special functions in the growth and development of plants. All the proteins found perform an essential role in mitochondria and chloroplast with the respect of photosynthesis and respiration. These entries consist of various proteins involved in cytochrome C assembly from plant mitochondria and bacteria; *CycK* from *Rhizobium leguminosarum* [PMID: 7665469], *CcmC* from Escherichia coli and *Paracoccus denitrificans* [PMID: 7635817, PMID: 9043133] and orf 240 from Wheat (*Triticum aestivum*) mitochondria [PMID: 7529870]. The members of this family are probably integral membrane proteins with six predicted trans-membrane helices that may comprise the membrane component of an ABC (ATP binding cassette) transporter complex. This transporter may be necessary for the transport of component needed for cytochrome C assembly. (http://www.ebi.ac.uk/interpro/entry/IPR002541).

## Discussion

In the present study, we have assessed the diversity of mtDNA and cpDNA in addition to the phylogenetic relationships between *Prunus* genotypes that might be helpful for identifying populations and their relationships [34]. Information on polymorphic DNA in organelle genomes is necessary for evolutionary investigations [23,35]. Though, it is demanding to perform high-throughput analysis on mitochondrial and chloroplast DNA polymorphisms [36,37]. Researchers in the past have used numerous non-coding cpDNA regions to obtain adequate characters for phylogenetic resolution [38-40]. At low taxonomic levels, some non-coding cpDNA regions might show sufficient variation for phylogenetic resolution while others did not [41,42]. The genetic diversity explained by SSR markers in the studied genotypes ranged from 0.35 to 0.85 which is quite acceptable. Our results showed that there is a complete association between Cherry and plum genotypes in both mtSSR and cpSSRs, though there is a low similarity index of Plum (L1 Tai Yang Li) with the rest of genotypes. These findings are in accordance with Moore and Ballington [12,43] who found that the cherry species *P. besseyi* and *P. pumila* are closely related to plums than to cherries. The cpDNA is inherited maternally in cherry and especially useful for phylogenetic studies due to its high degree of base sequence conservation [6,44]. Though it is highly conserved within species and shows higher rates of mutation in non-coding regions within the chloroplast genome.

In the present study, we detected single nucleotide polymorphism (SNPs) at the different levels in mitochondrial DNA sequence. We also observed some deletions and insertions in some points that might be due to mutations. As previously mentioned in other plant species, polymorphism is mostly based on insertions or deletions of single nucleotide A or T residues within mononucleotide sequences present in interspecific chloroplast genome regions [45].

In the case of cpSSR (ARCP511), microsatellites showed the harmonious amplicon length; however, no single nucleotide polymorphism were found, but deletions at various points were detected, such as deletions in CZI, CHO, CL1 and in CHU at 57, in CHU at 77, and in CY2, CL2 and in Cy1 at 131 were observed (Figure 2). Comparatively, high level of intra-group variations was found within genotypes. The present results depicted that regarding organelle DNA, microsatellite markers can be effectively useful for determining genetic diversity among the genotypes. Slight and intermediate size inversions are common features of the non-coding cpDNA [46] and are detectable only through sequencing and demonstrating intra-specific variability [47,48]. Microsatellites are widespread structures in non-coding cpDNA that became important population genetic markers [49].

The phylogenetic scope is correlated with the levels of genomic sequence divergence, defined in this context as the average number of nucleotide changes affecting neutral sites. We obtained high similarity among genotypes and variable levels of genetic dissimilarity in mtSSR and cpSSR with all tested microsatellite primer pairs [50]. We did not observe any genotype showing complete divergence form the rest of genotypes; however, there was low level of dissimilarity in both cp and mtSSR tested primers. Overall, in terms of size, organization and sequence, mtDNA is the most conservatively evolving genome. Alverson et al. [51] also found that the genetic similarity in organelle DNA and reported a strong relationship of genetic relation in Legumes. Most of the nuclear fragments confirmed correspondence to transposable elements, and one fragment harmonized that was previously found in the mitochondrial genomes. The MEHU (Huang Tao) showed diversity as compared to the rest of genotypes that might be due to the cross between Mai Huang Pan Tao and Binaced, non-Chinese landraces, which demonstrates separation of Chinese genotype from introduced genotypes. Previous researches on the evolutionary history of peach have also indicated a high probability that the Spanish non-melting peaches were evolved from northwest Chinese peaches [52,53].

Based on comparisons among all characterized land plants in previously published reports, woody species have a low cpDNA substitution rate, though a limited number of single nucleotide polymorphisms were detected [54,55]. Our study also demonstrated that cpDNA vary less than twofold in size, from 27 to 207 kb; moreover, almost two-third of the observed results of variations in sequence complexity, which varies only from 1 to 183 kbs in CY2, CL2 and CY1, but changes in the size of a large inverted repeat sequence, are present in almost all chloroplast genomes. These results are in agreement with Palmer et al. [9]. The conservation of polymorphism of locus among different botanical families suggests that it can stand a higher level of sequence variation within the chloroplast genome. Plastids are maternally inherited in most angiosperm species [56]. Chloroplast genomes are very stagnant in length and, large size mutations (additions and deletions) occurred rarely [57]. The small length mutations of a few bps to several hundred bps are relatively common during chloroplast genome evolution. The linear order and arrangement of chloroplast sequences is extremely conserved almost in all land plants. The genomic DNA sequences are valuable for resolving the plant phylogeny at deep levels of evolution because of their lower rates of silent nucleotide substitution [20]. Bortiri et al. [58] also reported that the sequence of *Prunus* species have many ambiguities and some of them are in the sequence regions with high variability. Additional studies including more accessions of *Prunus* species and more molecular data would be required to understand the genus and to draw a more precise phylogeny of *Prunus*.

In mitochondrial sequences, we found a highly conserved sequence almost in both SSR primers (CCB256 and NAD6), furthermore Satoh et al. [59] reported that sequences similar to nuclear DNA have also been reported to comprise 46.5% [60], 33% [51] and 13.4% of the total mtDNA length in melon, cucumber and rice, respectively [61]. Rodríguez-Moreno et al. [60] suggested that the extent of similarity indicates the massive induction of nuclear sequences into mitochondrial DNA; however, no attempts were made to determine the direction of sequence transfer. Similarly, Satoh et al. [59] made no efforts to assume the direction of the transfer for the 17.9% of the nuclear-like DNA within the unique mtDNA fraction [13,52].

The NCBI BLASTx based results of mtDNA and cpDNA sequences found three different proteins Cytochrome C assembly protein, PRKO 6433 super family and ndhk in mitochondrial and chloroplast, respectively. The previous work of Tsuji et al. [62] showed that the transcript abundance for some nuclear encoded subunits of cytochrome oxidase are oxygen-responsive and an oxia-suppressed, whereas the transcripts for mitochondrial encoded subunits are unaffected. Millar et al. [63] reported the abundance of cytochrome C that acts as an electron shuttle between complexes III and IV and was analyzed directly by antibodies raised from pigeon cytochrome C. Based on densitometry measurements, it was found that Cytochrome C protein increased more than 7-fold during air adaptation. Rice cytochrome C, oxidase complex and in the mitochondrial membrane in anoxic samples and the dramatic increase in the abundance of these complexes on air adaptation.

## Conclusion

These results provide the significance of organelle DNA diversity detected in species and within genotypes of *Prunus*. These findings also provide in depth understanding of the mitochondrial and chloroplast genomes. These results can also be used as fundamental data to begin detailed phylogenetic analysis of *Prunus* species. In addition these findings also provide new knowledge and the usefulness of cross-species transferability of microsatellite sequences allowing the discrimination of different genotypes of species with sequences developed in other species of the same genus.

## Methods

### Plant materials

The experimental material was consisted of seven genotypes, 2 from *Prunus avium* (Y2 Wn Ying and Y1 Hong Ying), 2 from *Prunus persica* (Huang Tao and Hong Tao), and 3 from *Prunus domestica* (L1 Tai Yang Li, L2 Hong Xin Li and Zi Ye Li) (Table 1). To carry out organelle (mtSSR and cpSSR) microsatellite marker analysis the above mentioned seven genotypes were collected from the experimental nursery at Jiangsu Province Institute of Botany, Nanjing and grown under standard cultivation conditions.

**Table 1:** **List of prunes genotypes analyzed**

**S. No.**	**Common name**	**Botanical name**	**Genotypes**	**Abbreviation**
1	Peach	*Prunus persica*	Huang Tao	HU
2	Peach	*Prunus persica*	Hong Tao	HO
3	Cherry	*Prunus avium*	Y1 Hong Ying	Y1
4	Cherry	*Prunus avium*	Y2 Wu Ying	Y2
5	Plum	*Prunus domestica*	L1 Tai Yang Li	L1
6	Plum	*Prunus domestica*	L2 Hong Xin Li	L2
7	Plum	*Prunus domestica*	Zi Ye Li	ZI

### Total DNA extraction and PCR amplification

The DNA was isolated from frozen leaves according to the Cetyl Trimethyl Ammonium Bromide (CTAB) method described by Cheng et al. [64] with slight modifications. DNA quality was examined by electrophoresis in 0.8% (w/v) agarose gels, and DNA concentration was quantified using a spectrophotometer. Extracted DNA was diluted to 100 ng/ul. Initially, amplifications were carried out by using two primer pairs of mtDNA and one for cpDNA (Table 2). DNA amplification was performed in a final volume of 25 uL containing 2.0 μl of DNA template, 2.5 μl of buffer, 1.5 mM of MgCl, 0.2 mM of dNTPs, 0.25 r Taq DNA polymerase and 0.2 μM of forward and reverse primers. Using Eppendorf thermo cycler (Eppendorf AG Hamburg, China), PCR was programmed as, 1 cycle of 4 min at 95°C, 35 cycles of 45 seconds at 94°C, 30 sec at different temperatures (annealing temperatures and extension times for each primer pair are provided in Table 2) and last cycle was followed by a final incubation for 10 min at 72°C. The PCR products were analyzed on polyacrylamide 8% gels and the gels were silver-stained according to the reported protocol [65,66] to detect the amplicon.

**Table 2:** **Pairs of mtDNA and cpDNA primers used for PCR-amplification and to obtained approximate PCR product size**

	**Code**	**Sequence**	**Temperature °C**	**Reference**
**Mitochondrial**	CCB256	GGAAGTTAGCAAAGTTAGAC	56	[67]
		TTGTTCTTAACAGCGATGGC		
	NAD6	TGAGTGGGTCWGTCGTCCTC	58	[67]
		TGATACTTTCTGTTTTGTCG		
**Chloroplast**	ARCP511	GGCCATAGGCTGGAAAGTCT	60	[68]
		GTTTATGCATGGCGAAAAGG		

### Organelle DNA extraction from PAGE

The PCR products were analyzed on denaturing polyacrylamide gels and the gels were silver-stained. For further purification and conformation of target band, approximate amplified fragments were extracted from the corresponding polyacrylamide gel. All the samples were crushed into pieces in 1.5 ml centrifuge tubes and incubated overnight with the high concentration of salt and buffer (MgCl_2_ 15 ul, buffer 12 ul and 100 ml ddH_2_O in each sample) at 37°C in water bath. The samples were centrifuged at 12000 rpm for 5 minutes, 150 μl of supernatant was mixed with 75% ethanol for DNA precipitation for 5 minutes and centrifuged at 12000 rpm for 5 min. The supernatant was discarded and pellets were air dried. After the extraction of organelle DNA, PCR products were re-amplified with the conservative primer pairs by using the same PCR programs as mentioned above. Amplified PCR products were separated on 2% (w/v) agarose gels, stained with ethidium bromide, and visualized under ultraviolet (UV) light. The approximate size of amplified fragments was estimated with a 1-kb ladder DNA marker (Takara). The target bands were excised and extracted using the DNA gel extraction kit, (AXYGEN Bioscience, China) according to the manufacturer’s protocol.

### TA cloning and sequencing

The eluted DNA fragments were hydro sheared, cloned and DNA samples obtained were ligated into the pMD 19-T Vector (Takara Biotech, Dalian, China) for 9 hours at 16°C and transformed into *Escherichia coli* strain DH5α. At least three positive fragments were sequenced by combining 2 independent PCR amplicon. The DNA was automated sequenced by the INVITROGEN Company (Shanghai, China).

The multiple sequence alignment was conducted by ClustalX 1.83 programs, and the phylogenetic relationships were inferred by using the Neighbor-Joining (NJ) method with 1000 bootstraps in MEGA 4.0.1 software.

## Availability of supporting data

### GenBank accession numbers

#### Primer CCB256:

The nucleotide sequence data reported in this paper will appear in the GenBank nucleotide sequence databases with the following accession numbers:

*Prunus persica*; HuangTao, KM878736; HongTao, KM878737

*Prunus avium*; Y1HongYing, KM878738; Y2WuYing, KM878739

*Prunus domestica*; L1TaiYangLi, KM878740; L2HongXinLi, KM878741; ZiYeLi, KM878742

#### Primer NAD6:

*Prunus persica*; HuangTao KM878743; HongTao KM878744

*Prunus avium*; Y1HongYing KM878745; Y2WuYing KM878746

*Prunus domestica*; L1TaiYangLi KM878747; L2HongXinLi KM878748; ZiYeLi KM878749

### Voucher specimens

Voucher specimens were gathered from the leaf specimens collected at 15 July 2013, from the Experimental nursery at Jiangsu Province Institute of Botany, Nanjing, China (GPS coordination: latitude 32° 05, longitude 118° 83), and identified by Y. Hong and T. Yang.
